# A PIF‐regulated switch in cell axis growth drives cotyledon expansion through tissue‐specific cell expansion and division

**DOI:** 10.1111/tpj.70196

**Published:** 2025-05-16

**Authors:** Nil Veciana, Guiomar Martín, Elena Monte

**Affiliations:** ^1^ Centre for Research in Agricultural Genomics (CRAG) CSIC‐IRTA‐UAB‐UB, Campus UAB Bellaterra Barcelona Spain; ^2^ Department of Biology, Healthcare and the Environment, Faculty of Pharmacy and Food Sciences University of Barcelona Barcelona 08028 Spain; ^3^ Consejo Superior de Investigaciones Científicas (CSIC) Barcelona Spain

**Keywords:** cotyledon expansion, light, PIFs, phytochromes, GUN1, retrograde signaling, epidermis, palisade, cell expansion and division, *Arabidopsis thaliana*

## Abstract

Despite its crucial role during seedling deetiolation, cotyledon expansion has been largely overlooked, with hypocotyl elongation favored as the primary phenotypic readout in light signaling research. Here, we investigate how cotyledon expansion is regulated during seedling establishment and reveal that light‐induced cotyledon expansion involves a rapid switch in growth direction – from longitudinal in darkness to transversal upon initial light exposure. Using PIFq‐ and phyA/phyB‐deficient Arabidopsis mutants, we demonstrate that this switch is repressed by PIFs in the dark and promoted by phytochromes under red light. Notably, expansion is antagonistically regulated in the light by GUN1‐mediated plastid retrograde signaling. Cotyledon expansion involves rapid epidermis cell expansion, transitioning from rectangular in darkness to characteristic lobed cells in light. Importantly, our findings show that mesophyll extension is driven not only by cell enlargement but also by palisade cell division, consistent with an enrichment of cell cycle‐related genes that are antagonistically regulated by the PIF/phy system and retrograde signaling in the cotyledon. Finally, using mutant lines expressing PIF1 and phyB specifically in the epidermis, we establish that epidermal expansion can drive palisade cell growth, while mesophyll cell division is predominantly regulated by light at the tissue‐specific level. This study provides a novel framework for investigating cotyledon expansion during seedling deetiolation, incorporating tissue‐level regulation. We propose that cotyledons serve as an excellent model for studying morphogenesis and organ geometry, which in plants is governed by directional cell growth.

## Significance Statement

Despite their critical role in the successful establishment of green photoautotrophic organisms, the mechanisms governing cotyledon expansion during seedling deetiolation remain largely unknown. Our study reveals that the phy/PIF system drives a light‐induced shift in the cotyledon growth axis to promote cotyledon growth through tissue‐specific cell expansion and division, while GUN1‐mediated plastid signals counteract this process when chloroplast biogenesis is impaired.

## INTRODUCTION

Cotyledons are embryonic leaves vital for seedling establishment and the early stages of plant development after germination (Wang et al., 2019). In Arabidopsis, they are rich in lipids and serve as a nutrient source to fuel seedling development, including the differentiation of the cotyledon embryonic proplastids. If germination occurs in the dark, seedlings undergo skotomorphogenesis or etiolation, exhibiting a fast‐growing hypocotyl, a hook to protect the apical meristem, and closed unexpanded cotyledons containing etioplasts. Exposure to light triggers photomorphogenesis or de‐etiolation, characterized by extensive transcriptional reprogramming (Jiao et al., 2007) that drives chloroplast development and inhibition of hypocotyl growth in synchrony with the unfolding and expansion of the cotyledons that become the major photosynthetic tissue, a switch to autotrophy critical for seedling survival (Gommers & Monte, 2018).

Photomorphogenesis is initiated by a suite of photoreceptors. Among them, the phytochromes (phy) perceive light of the red and far red wavelengths (600–750 nm). The phy family contains five members in Arabidopsis, the photolabile phyA and the photostable phyB–phyE (Sharrock & Quail, 1989). Because phyA is the most abundant phytochrome in dark‐grown seedlings (Debrieux & Fankhauser, 2010), and phyB is the most abundant and the main contributor in the light (Cantón & Quail, 1999), the concerted action of phyA and phyB regulates the majority of the responses of dark‐grown seedlings when first exposed to red light (Tepperman et al., 2006). Phys exist in two reversible conformations: the inactive Pr form is synthesized in the cytosol, and absorbs red light to photoconvert to the active Pfr form. Pfr inactivates back to Pr when absorbing far‐red light or in prolonged darkness (Ulijasz et al., 2010). Upon activation, phy Pfr translocates to the nucleus where it interacts with the basic helix–loop–helix type transcriptional regulators PHYTOCHROME INTERACTING FACTORS (PIFs) (Ni et al., 1999). PIFs accumulate in the dark and promote skotomorphogenesis through regulation of about 10% of the genes in the genome (Leivar et al., 2008, 2009; Leivar & Monte, 2014; Monte et al., 2004). In the light, PIF interaction with active phy Pfr interferes with their DNA binding and induces their rapid phosphorylation and degradation, modifying the gene expression landscape and allowing photomorphogenesis to proceed (Leivar et al., 2008, 2009; Monte et al., 2004; Park et al., 2012). The importance of PIFs as photomorphogenesis repressors during post‐germinative development in the dark is manifested by the constitutive photomorphogenic (cop)‐like phenotype of the PIF quadruple Arabidopsis mutant *pifq*, which lacks the PIF quartet (PIFq) (PIF1, PIF3, PIF4, and PIF5) and displays short hypocotyl, opened apical hook, and unfolded and expanded cotyledons in the dark (Leivar et al., 2009; Shin et al., 2009). Light‐activated phytochromes also inhibit the COP1–SPA complex, which stabilizes HY5 and other photomorphogenesis‐promoting factors (Sheerin et al., 2015; Wang et al., 2021).

Photomorphogenesis can also be regulated by chloroplast‐to‐nucleus retrograde signaling (RS), which can converge with phy signaling to antagonistically regulate seedling development (Martin et al., 2016). Seedlings treated with lincomycin or norflurazon (inhibitors of chloroplast biogenesis) display closed, unexpanded cotyledons in the light, resembling dark‐grown seedlings (Martin et al., 2016; Ruckle & Larkin, 2009). RS‐induced repression of photomorphogenesis is mediated by GENOMES UNCOUPLED 1 (GUN1) (Martin et al., 2016; Ruckle & Larkin, 2009) and is independent of the PIFq (Martin et al., 2016). RS and phy signaling converge to regulate *GLK1*, and the GUN1/GLK1 module inhibits the PIF‐repressed transcriptional network to suppress cotyledon development when chloroplast integrity is compromised (Martin et al., 2016), at least in part through the regulation of BBX16 (Veciana et al., 2022).

Cotyledons are structured as stacked combinations of cell layers (Stoynova‐Bakalova et al., 2004). In the light, the epidermis (upper or adaxial, and lower or abaxial) is characterized by the pavement cells in a characteristic jigsaw puzzle arrangement (Carter et al., 2017; Lin et al., 2015) and the embedded stomata (Bean et al., 2002; Chen et al., 2012; Rovira et al., 2024). Between the two epidermises, the mesophyll is composed of two distinct cell layers: the palisade under the adaxial epidermis, consisting of tightly packed column‐shaped cylindrical cells (in perpendicular orientation to the cotyledon blade plane) that contain large amounts of chloroplasts and are the main contributor to photosynthesis (Gotoh et al., 2018; Outlaw et al., 1976); and the spongy mesophyll between the palisade and the abaxial epidermis, conformed by loosely organized spherical‐shaped cells with large air pockets within them and fewer chloroplasts, with the primary function of allowing gas flow within the cotyledon/leaf to facilitate photosynthesis (Borsuk et al., 2022).

How cotyledon expansion occurs is relatively underexplored. Based on their mutant phenotypes, a role for the phy‐PIF module in light‐induced cotyledon expansion has been established (Leivar et al., 2009; Neff & Van Volkenburgh, 1994; Shi et al., 2018; Shin et al., 2009), yet how this is achieved is still mostly unknown. Previous work has proposed that it is largely a consequence of cell expansion rather than cell division (Neff & Van Volkenburgh, 1994; Stoynova‐Bakalova et al., 2004), but the relative contribution of each process and the cell layers involved are still open questions in the field. Numerous studies have shown the expansion dynamics of epidermis cells in dark‐grown seedlings exposed to light, from the increase in size to the characteristic lobe and jigsaw puzzle formation of the pavement cells (Carter et al., 2017; Higaki et al., 2016; Lin et al., 2015). In contrast, cell division in the epidermis has been mostly conducted around stomatal development, where epidermal cells are produced as division byproducts from stomatal precursors (Houbaert et al., 2018; Smit et al., 2023). The contribution of this process to the overall cotyledon expansion is unlikely to be significant. In contrast to the epidermis, research on cotyledon mesophyll concerning cotyledon expansion is limited (Stoynova‐Bakalova et al., 2004).

Here, we examine cotyledon expansion in the dark and address the effect of light during deetiolation, focusing on the contribution of the epidermis and the palisade cell layers. Our results indicate that the phy/PIF system regulates a switch in the axis growth necessary for cotyledon expansion, which is antagonistically regulated by GUN1‐mediated RS. Moreover, we show that cotyledon expansion involves not only light‐induced cell expansion in the epidermis and the palisade, but also PIF‐regulated mesophyll cell division. Using lines expressing epidermis‐specific PIF1 and phyB, our data suggest a model whereby the epidermis drives cotyledon expansion supported by tissue‐autonomous palisade cell division regulated by the phy/PIF system.

## RESULTS

### Cotyledon expansion during de‐etiolation involves a light‐induced switch in the growth axis

**Figure 1:**
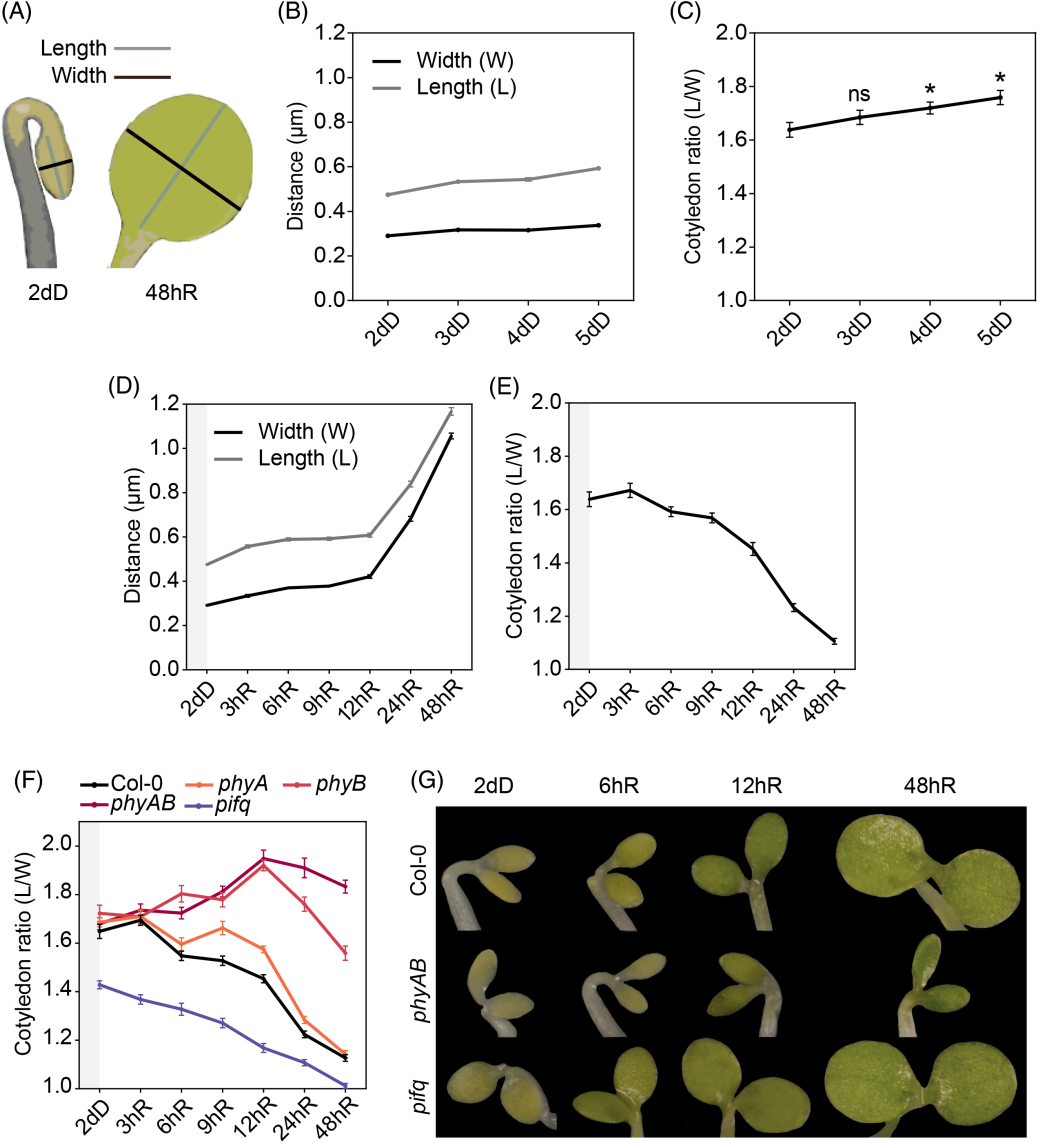
The PHY‐PIF module regulates cotyledon expansion and shape during de‐etiolation. (A) Etiolated (left) and de‐etiolated (right) cotyledons with length (gray) and width (black) axes indicated. (B) Characterization of cotyledon growth in the two axes in seedlings grown for 2, 3, 4, and 5 days in the dark (dD). (C) Quantification of cotyledon ratio (length/width) in WT seedlings grown in (B). Data are means ± SEM of at least 25 seedlings. Statistical differences between mean values at each time point compared with 2dD were analyzed by Student's *t*‐test (*P* < 0.05) and indicated with an asterisk. ns, non‐significant. (D) Characterization of cotyledon growth during de‐etiolation in the length and width axes. WT seedlings were grown for 2 days in the dark (2dD) and transferred to continuous red light (R) for 3, 6, 9, 12, 24, and 48 h. (E) Quantification of cotyledon ratio in WT seedlings during de‐etiolation. Data are means ± SEM of at least 25 seedlings. (F) Quantification of cotyledon ratio in Col‐0, *pifq*, *phyA*, *phyB*, and *phyAB* seedlings grown for 2 days in the dark (2dD) and transferred to continuous red light (R) for 3, 6, 9, 12, 24, and 48 h. Data are means ± SEM of at least 20 seedlings. (G) Visual phenotypes of representative Col‐0, *phyAB*, and *pifq* cotyledons during de‐etiolation at selected time points.

To assess how cotyledon expansion takes place during deetiolation, we measured cotyledon blade growth in the length and width (at the maximum point) axis, and used cotyledon ratio (length of the cotyledon divided by the width, or L/W) as described (Neff & Van Volkenburgh, 1994) to capture the bidirectional expansion of the cotyledon surface. In 2‐day‐old dark‐grown Arabidopsis seedlings (2dD), cotyledons were longer than wider, displaying an elliptical shape with a ratio value of ~1.6 (Figure 1A–C). If grown in the dark for longer, the width was maintained unaltered during at least the following 3 days, whereas the length progressively increased, leading to an increase in the ratio in the dark from 1.6 to ~1.8 (Figure 1B,C). In contrast, if 2dD seedlings were exposed to red light, there was a switch in the preferred growth axis especially apparent after 12 h, and this activation of growth in the width axis led to a perfectly round cotyledon (ratio = 1 after 48 h of continuous red light) (Figure 1A,D,E) and an increase in the area of ~7‐fold (from 0.13 mm^2^ in 2dD to 0.9 mm^2^ in 48hR). Together, these observations suggest that light triggers a fast switch in the preferential axis growth of the cotyledon (from length to width) allowing expansion to reach a round cotyledon shape when seedlings are exposed to light.

### The PHY‐PIF module promotes the light‐induced axis growth switch

To start to understand how expansion in the two axes is regulated, we next performed a de‐etiolation experiment using *phyA*, *phyB*, *phyAB*, and *pifq*. In 2dD seedlings, none of the three *phy* mutant lines showed any difference in cotyledon ratio compared with Col‐0 (WT) (~1.6). In contrast, *pifq* displayed a lower ratio of ~1.4, as expected given their expanded cotyledon phenotype in the dark (Figure 1F) (Leivar et al., 2009; Shin et al., 2009). Upon red‐light exposure, *phyA* mutants exhibited delayed cotyledon expansion during the first hours (9–12 h) but eventually resumed growth until the cotyledon ratio reached WT‐like levels after 48 h of red‐light treatment (~1.1). Remarkably, in *phyB* and *phyAB* mutants, cotyledons not only did not reduce their length/width cotyledon ratio, but the ratio increased in red light (Figure 1F,G) (~1.9 after 24 h of red), revealing a lack of the width‐growth priority and a continuous growth in the length axis, mimicking the behavior observed in dark cotyledons (Figure 1C). The repression of expansion was not sustained in *phyB –* eventually expanded after 48 h – but was maintained in *phyAB* after 2 days of red light (Figure 1F,G), in accordance with phyA and phyB acting in concert during deetiolation (Tepperman et al., 2006). These results support the idea that cotyledon expansion requires a light‐induced switch in the preferential axis growth of the cotyledon and indicate that this switch is repressed by PIFs in the dark and is activated by phy signaling during the first hours of light.

### Convergence of phy/PIF and RS in the regulation of cotyledon expansion

**Figure 2:**
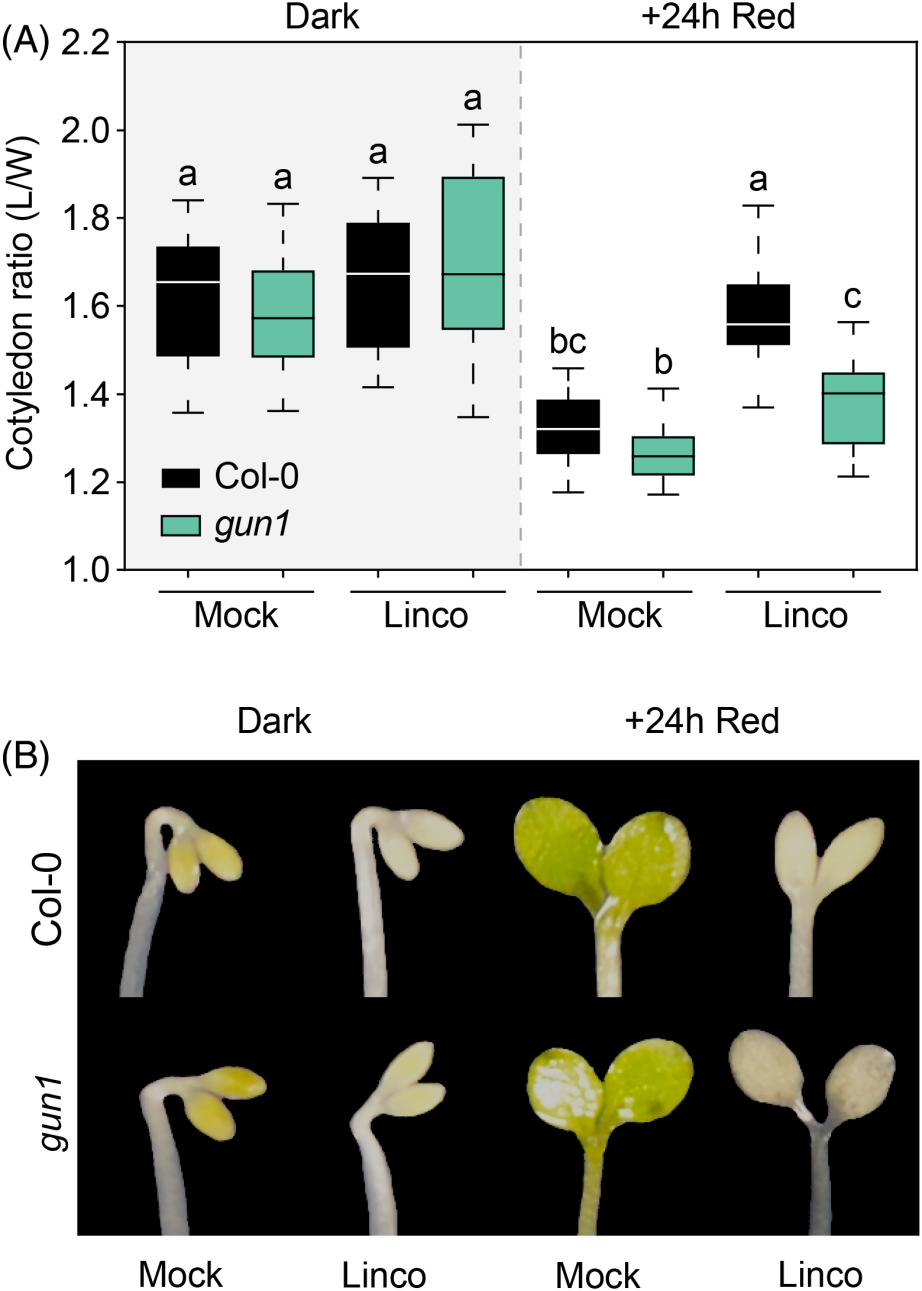
GUN1‐mediated retrograde signaling regulates cotyledon expansion and shape during de‐etiolation. (A) Cotyledon ratio (length/width) quantification of WT Col‐0 and *gun1* seedlings grown for 2 days in the dark and transferred to red light for 24 h, without (mock) or with (Linco) lincomycin. Data in boxplots indicate the first quartile, median, and third quartile of *n* ≥ 20 seedlings. Whiskers indicate the 5 to 95 percentile. Letters denote the statistically significant differences using two‐way ANOVA followed by *post hoc* Tukey's test (*P* < 0.05). (B) Visual phenotypes of representative Col‐0 and *gun1* cotyledons of seedlings grown as in (A).

GUN1‐mediated inhibition of cotyledon expansion upon chloroplast disruption in continuous light has been described before (Martin et al., 2016; Ruckle & Larkin, 2009). However, whether GUN1‐mediated RS also affects cotyledon expansion in the dark and/or in dark‐grown seedlings when first exposed to light remains to be determined. We first evaluated how lincomycin affected cotyledon ratio in 2dD WT seedlings. Compared with non‐treated seedlings, the ratio was similar (~1.6) (Figure 2A,B). However, upon red light exposure for 24 h, whereas non‐treated cotyledons expanded to a ratio of ~1.35, lincomycin inhibited WT cotyledon expansion and the ratio remained at ~1.6 (Figure 2A,B). Similarly to WT, *gun1* cotyledon expansion was unaffected by lincomycin in the dark. In contrast, upon transfer to red light, *gun1* cotyledons expanded even in the presence of lincomycin, and exhibited similar reduced ratios compared with dark‐grown *gun1* in non‐treated (~1.25) and treated (~1.4) conditions (Figure 2A,B). These results are in agreement with the previously described GUN1 accumulation in cotyledons of dark‐ and light‐grown as well as in deetiolating seedlings (Hernández‐Verdeja et al., 2022; Wu et al., 2018), and together they show that chloroplast disruption suppresses cotyledon expansion in dark‐grown seedlings during the first hours of red light exposure, maintaining etiolated‐like cotyledons. This suppression of photomorphogenesis by RS is GUN1‐mediated and indicates that the previously described antagonistic convergence of light and RS shown under continuous light conditions (Martin et al., 2016) also regulates cotyledon development during dark‐to‐light deetiolation. Interestingly, cotyledon expansion can proceed with a damaged chloroplast in the *gun1* mutant, suggesting that expansion can be fueled by the remaining seed energy in the cotyledons, and indicating that the lack of expansion in lincomycin‐treated WT is likely not due to a lack of chloroplast‐derived nutrients but rather a consequence of active chloroplast‐to‐nucleus signaling mediated by GUN1.

### The cotyledon expression network is antagonistically regulated by light and retrograde signals with an enrichment in cell division‐related genes

**Figure 3:**
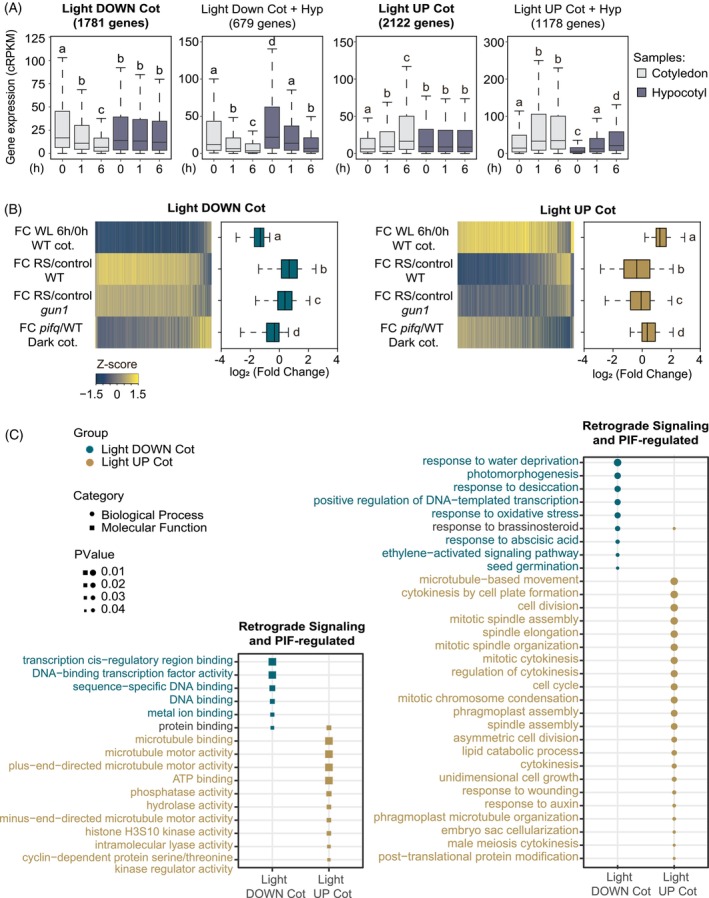
The cotyledon expression network is antagonistically regulated by phytochrome and retrograde signals with an enrichment in cell cycle genes. (A) Expression of genes in the “Light UP Cot,” “Light UP Cot + Hyp,” “Light DOWN Cot” and “Light DOWN Cot + Hyp” subsets in cotyledons or hypocotyls of dark‐grown WT seedlings exposed to 1 or 6 h of white light (WL). Letters denote statistically significant differences using one‐way Kruskal–Wallis test (*P* < 0.05). (B) Norflurazon antagonistically regulates the light‐induced and PIF‐repressed (left) and the light‐ repressed and PIF‐induced (right) networks of “Light UP Cot” and “Light DOWN Cot” gene sets, in a partially GUN1‐dependent manner. Heatmaps depict transformed *Z* score of fold‐change (FC) expression of 2122 genes (left) and 1781 genes (right) in WT cotyledons after 6 h of light exposure (top row), in WT and *gun1* after treatment with norflurazon (two middle rows), and in cotyledons of dark‐grown *pifq* compared with WT (bottom row). FC expression of the same gene sets is represented in boxplots. Letters denote statistically significant differences using one‐way Kruskal–Wallis test (*P* < 0.05). (C) Gene ontology terms of the “Light UP Cot” and “Light DOWN Cot” genes affected by at least twofold both in *pifq* in the dark (Sun et al., 2016) and by norflurazon in the light (Zhao et al., 2019). About 10% of the “Light UP Cot” gene set (219 of 2122 genes) and 6.5% of the “Light DOWN Cot” (117 of 1781 genes) met both criteria. (A, B) Gene expression values were quantified from raw data submitted at the Sequence Read Archive under the accession numbers GSE79576 (Sun et al., 2016) and GSE110125 (Zhao et al., 2019).

To start to understand the underlying transcriptional network involved in cotyledon expansion in response to light and retrograde signaling, we first took advantage of a previous report that described light‐regulated genes in dark‐grown seedlings exposed to light separately in cotyledon and hypocotyl, with a twofold change threshold (Sun et al., 2016). We reasoned that among these genes, significant candidate genes would likely belong to categories encompassing light regulated genes in cotyledon that are not affected in hypocotyl, and genes that are expressed in opposite direction in these two organs given the promoting and inhibiting effect of light in cotyledon and hypocotyl cell expansion, respectively (Bou‐Torrent et al., 2008; Martín & Duque, 2021). Accordingly, we defined two groups (see Experimental Procedures): light upregulated in cotyledons (Light UP Cot) (2122 genes in total, 1810 cotyledon specific and 312 also downregulated in hypocotyls), and light downregulated in cotyledons (Light DOWN Cot) (1781 genes in total, 1640 cotyledon specific and 141 also upregulated in hypocotyls). In contrast, we considered two additional groups containing light up‐ or light downregulated genes in both cotyledons and hypocotyls (Light UP Cot + Hyp, 1178 genes; Light DOWN Cot + Hyp, 679 genes) (Table S1) (see “Experimental procedures” section) as less likely to be involved in the regulation of cotyledon expansion. The gene expression for each of these four gene sets in the dark and upon light exposure in cotyledon and hypocotyl is shown in Figure 3(A) (values correspond to our mRNA quantification of sequencing data in Sun et al., 2016) (see “Experimental procedures” section). Next, we examined the expression levels of the “Light UP Cot” and “Light DOWN Cot” gene subsets in *pifq* in the dark and upon treatment with RS‐inducing norflurazon (NF), re‐quantifying mRNA sequencing data from Sun et al. (2016) and Zhao et al. (2019), respectively, for homogeneity (see “Experimental procedures” section). Our results indicate that the light‐regulated genes in cotyledon show a similar trend in expression in *pifq* in the dark (Figure 3B), in accordance with their regulation by the phy/PIF system as previously concluded (Sun et al., 2016). Finally, to evaluate their regulation by RS, we compared their expression upon treatment with NF (Zhao et al., 2019). The light‐regulated molecular phenotype in both the “Light UP Cot” and “Light DOWN Cot” gene subsets was strongly reversed when RS was activated (Figure 3B). Importantly, this reversion was partially dependent on GUN1 (Figure 3B) (Zhao et al., 2019).

Together, this analysis reveals that light activates a transcriptional program to reverse the PIFq‐imposed gene expression landscape in dark‐grown cotyledons, concomitant with the light‐promoted switch in cotyledon growth axis and their expansion. Importantly, activation of GUN1‐mediated RS antagonizes the effect of light, targeting both light‐induced and repressed gene networks, in agreement with the RS inhibition of cotyledon expansion in the light. We conclude that phytochrome and GUN1‐mediated RS antagonistically regulate gene expression in cotyledons.

Next, to identify the most significant functional categories in each of the “Light Cot” and “Light Cot + Hyp” groups, we identified the genes in each subset that were previously defined as PIFq‐regulated in the dark (Sun et al., 2016) and RS‐regulated in the light (Zhao et al., 2019). About 10% of the “Light UP Cot” gene set (219 of 2122 genes) and 6.5% of the “Light DOWN Cot” (117 of 1781 genes) met both criteria (Table S1). Gene Ontology analysis revealed that the “Light UP Cot” subset was highly enriched in cell cycle and cell division genes, in accordance with the previous results for the PIFs (Zhang et al., 2021), whereas categories enriched in the “Light DOWN Cot” subset included transcription factor activity and hormone related genes among others (Figure 3C). The specificity of these categories in the cotyledon is underlined by the differential gene ontology profile of the “Light UP Cot + Hyp” and “Light DOWN Cot + Hyp” subsets (Figure S1). Interestingly, these results suggest that cotyledon expansion might involve cell division, a long standing question in the field.

### Cotyledon epidermis growth during deetiolation relies solely on cell expansion

**Figure 4:**
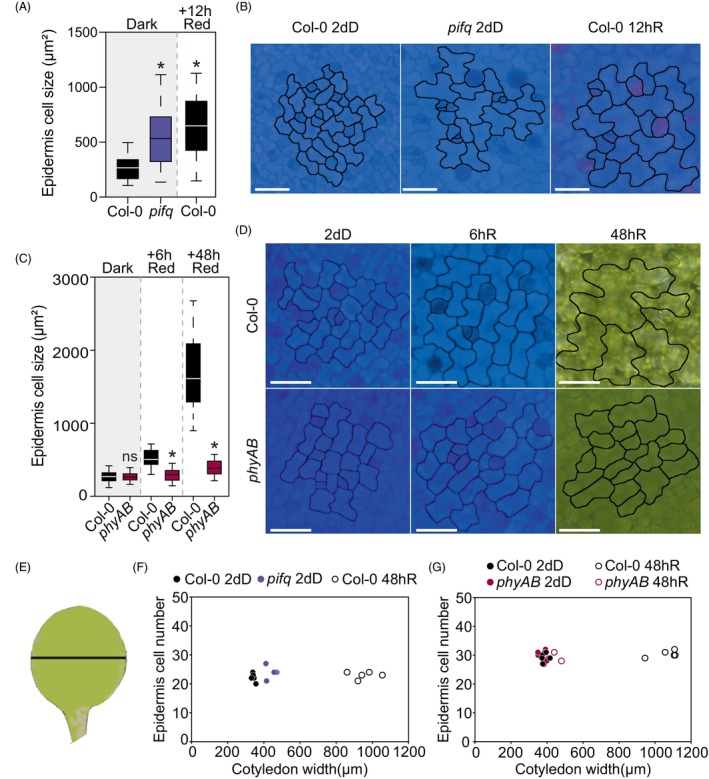
The PHY‐PIF module regulates light‐induced epidermis cell expansion during de‐etiolation. (A) Quantification of epidermis cell size in WT and *pifq* in 2‐day‐old seedlings (2dD) and WT seedlings transferred to red light (R) for 12 h. (B) Visual phenotypes of WT and *pifq* epidermis cells. (C) Quantification of epidermis cell size in WT and *phyAB* in 2‐day‐old dark seedlings (2dD) and transferred to continuous red light (R) for 6 and 48 h. (D) Visual phenotypes of WT and *phyAB* epidermis cells in de‐etiolation. Data in boxplots (A, C) indicate the first quartile, median, and third quartile of *n* ≥ 125 cells pooled from at least five individual cotyledons. Whiskers indicate the 5–95 percentile. Statistical differences relative to WT in each time point are indicated by an asterisk (Student's *t*‐test. *P* < 0.05). ns, non‐significant. (E) Representation of epidermis cell number relative to cotyledon width as shown by a black line in WT and *pifq* (F), and in WT and *phyAB* (G) seedlings grown in the dark for 2 days (2dD) and transferred to red light (R) for 48 h. Each dot represents an individual cotyledon.

We next sought to elucidate the contribution of cell expansion and division within the light‐induced expansion of the adaxial epidermis. In WT cotyledons grown in darkness, the epidermal pavement cells appeared elongated without lobes and aligned with the cotyledon long axis and shape (Figure 4A,B). After transferring to light, cells lost the preferential elongated growth and formed their characteristic lobes. After 2 days of red light, WT epidermis cells increased their size by 6.5‐fold compared with 2‐day‐old dark cells (Figure 4A–D). Remarkably, epidermis pavement cells in dark‐grown *pifq* seedlings were expanded and lobed, resembling cells in deetiolated WT cotyledons after 12 h of red light (Figure 4A,B). This was also observed in dark‐grown *cop1* seedlings (Figure S2), which exhibit lower levels of the PIFs in the dark (Pham et al., 2018). In contrast, in the double mutant *phyAB*, pavement cells in the dark were similar to WT but were unable to expand in red light, maintaining the dark cell morphology of long cells without lobe formation (Figure 4C,D). We did detect some residual growth, likely due to the activity of phyC‐E, in accordance with the lack of cotyledon expansion in seedlings deficient in all five phys (Hu et al., 2013).

With regard to cell division, we only visually observed dividing cells in the relatively smaller subsets of cells surrounding stomata, probably byproducts of stomata formation as described (Houbaert et al., 2018; Smit et al., 2023). To provide quantitative evidence and confirm the absence of cell division in the rest of the pavement cells, and given the major importance of width expansion as discussed above, we counted the number of cells present at the point of maximum width in the width axis of cotyledons, in a straight line from one edge of the cotyledon to the other, in 2dD seedlings and after 48 h of exposure to Red light (48hR) (Figure 4E–G). Next, for each measured cotyledon, we plotted the width in relation to the number of total cells in the width axis. Remarkably, despite the WT cotyledon width increase from approximately 400 μm in the dark to almost 1200 μm after 48 h of red light, the number of epidermal cells remains similar (Figure 4F). Dark‐grown *pifq* also showed no difference in the number of cells compared with 2dD or 48hR WT (Figure 4F), despite the wider and expanded *pifq* cotyledons in the dark (Figure 1). Similarly, we did not observe any changes in cell number in 2dD *phyAB* compared with 2dD WT or to 48hR WT (Figure 4G). Together, these results suggest that cell expansion rather than division is the main contributor to epidermis development during de‐etiolation, and that PIFs act as repressors of pavement cell expansion in the dark, a repression that is lifted upon exposure to red light through phy action.

### The palisade mesophyll experiences cell expansion and division during de‐etiolation

**Figure 5:**
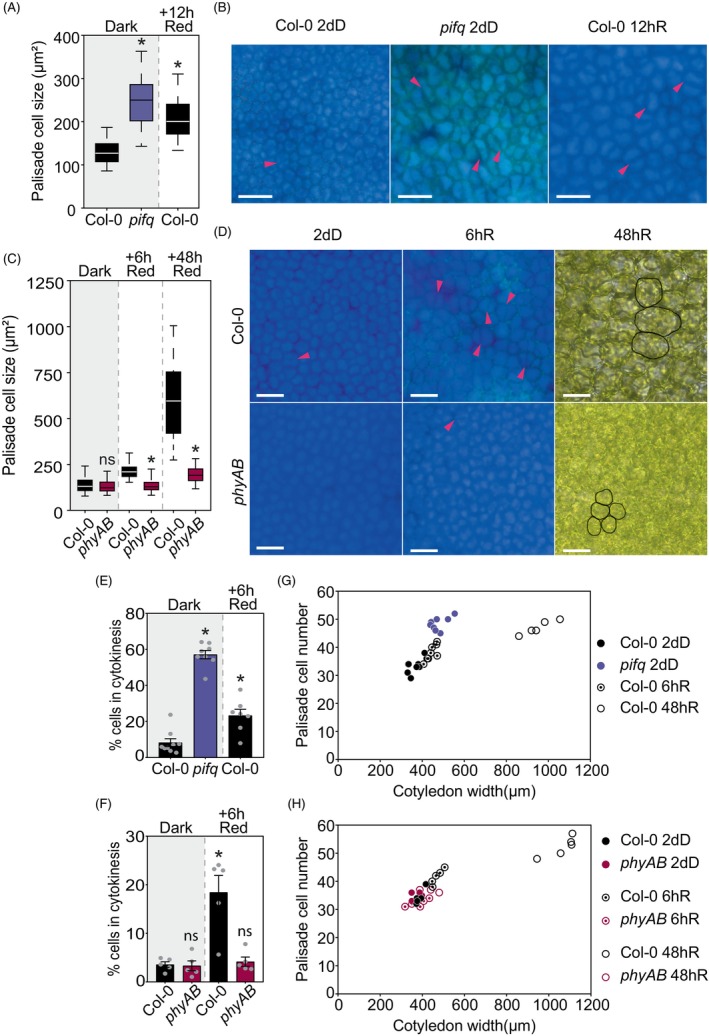
The PHY‐PIF module regulates the light‐induced palisade mesophyll cell growth during de‐etiolation. (A) Quantification of palisade cell size in WT and *pifq* in 2‐day‐old seedlings (2dD) and WT seedlings transferred to red‐light (R) for 12 h. (B) Visual phenotypes of WT and *pifq* palisade cells. (C) Quantification of palisade cell size in WT and *phyAB* in 2‐day‐old dark seedlings (2dD) and transferred to continuous red‐light (R) for 6 and 48 h. (D) Visual phenotypes of WT and *phyAB* palisade cells in de‐etiolation. (A, C) Data in boxplots indicate the first quartile, median, and third quartile of *n* ≥ 125 cells pooled from at least five individual cotyledons. Whiskers indicate 5–95 percentile. Statistical differences relative to WT in each timepoint are indicated by an asterisk (Student's *t*‐test, *P* < 0.05). ns, non‐significant. (B, D) Magenta arrows indicate visible cell division. (E) Percentage of palisade cells with a visible division plane in WT and *pifq* in 2‐day dark‐grown seedlings (2dD) and WT seedlings transferred to red light (R) for 6 h. (F) Percentage of palisade cells with a visible division plane in WT and *phyAB* in 2‐day dark‐grown seedlings (2dD) and transferred to red light (R) for 6 h. (G) Representation of palisade cell number relative to cotyledon width in WT and *pifq* seedlings grown in the dark for 2 days (2dD) and WT seedlings transferred to red light (R) for 6 and 48 h. (H) Representation of palisade cell number relative to cotyledon width in WT and *phyAB* seedlings grown in the dark for 2 days (2dD) and transferred to red light (R) for 6 and 48 h. Data in (E, F) indicate means ± SEM of at least six cotyledons. Asterisks indicate statistically significant differences relative to WT 2dD using Student's *t*‐test (*P* < 0.05). In (G, H), each dot represents an individual cotyledon.

Palisade cells, oriented with their long axis perpendicular to the cotyledon blade plane, can be visualized as circular structures in top‐view microscopy in dark‐ and light‐grown seedlings. In deetiolating WT, palisade cells rapidly expanded, from approximately 150 in the dark to approximately 240 μm^2^ after 12 h (Figure 5A,B), and to 600 μm^2^ after 48 h, a size increase of 4.5‐fold (Figure 5C,D). In contrast, in dark‐grown *pifq*, palisade cells were clearly larger and displayed a size comparable to WT cells after 12 h of red light (Figure 5A,B). In *phyAB*, palisade cell size in the dark was similar to WT, but the red‐light‐induced expansion was strongly inhibited, showing only a slight growth after 48 h (~220 μm^2^) (Figure 5C,D). These results resemble the expansion dynamics of the epidermis and the impact of the mutations in PIFq and phyA/B. However, in contrast to the epidermis, light induces palisade cell expansion without significantly changing the morphology (Figure 5B,D).

Interestingly, we visually observed dividing cells after the first hours of light. Indeed, a fraction of the cell population showed a clear cell wall division plane that divides the otherwise spherical palisade cell into two roughly identical cells (Figure 5B,D). Accordingly, the fact that deetiolating epidermis cells increase on average 6.5‐fold in size compared with the 4.5‐fold of palisade cells supports the notion that palisade cells must undergo division to support synchronized growth. To provide quantitative evidence, we assessed the percentage of visible palisade cells in cytokinesis in 2dD seedlings and upon red light exposure, in a centered square of 50 × 10^3^ μm^2^ that covered most of the cotyledon area. In WT seedlings, approximately 5% of the palisade cells in the dark showed a visible division plane, that rapidly increased to approximately 20% after 6 h of red light (Figure 5E). Remarkably, in *pifq*, the percentage of visible cells in cytokinesis in the dark was above 50% (Figure 5E). Increased % of visible cells in cytokinesis was also observed in dark‐grown *cop1* seedlings (Figure S2), whereas in *phyAB* mutant seedlings the % of pavement cells was similar to WT in the dark and did not increase upon red light exposure (Figure 5F). Next, using the same approach as in the epidermis, we counted the number of cells in the width axis in different conditions and genotypes. Unlike the epidermis, the number of palisade cells in WT cotyledons increased from approximately 35–40 cells in the dark, to approximately 45 after 6 h of red light, and approximately 50–55 after 48 h, an increase of approximately 1.4× compared with dark (Figure 5G). Remarkably, in 2dD *pifq*, as a result of the increased cell division (Figure 5E), the number of total cells was similar to 2dD WT exposed to 48 h of red light (Figure 5G). In the *phyAB* mutant, the lack of cotyledon expansion in red light was reflected in the number of palisade cells, which remained the same and similar to WT in the dark and after 48 h of red light (Figure 5H). Together, these results indicate that during de‐etiolation, cotyledon expansion involves division and expansion of palisade mesophyll cells, a process that is repressed by PIFs in the dark and activated upon exposure to red light through phy activity. PIF repression of palisade cell division is in accord with previous results showing PIF regulation of cytokinesis‐related genes in the cotyledons (Zhang et al., 2021). Importantly, these results open the intriguing question of whether the increase in palisade cell division during deetiolation is induced by a PIF/phy‐mediated light signal, and/or triggered by a mechanical stress signal due to the expansion forces of the adjacent cells, including the epidermal.

### Regulation of cotyledon expansion by cell‐autonomous PIF and phyB activity in the epidermis

**Figure 6:**
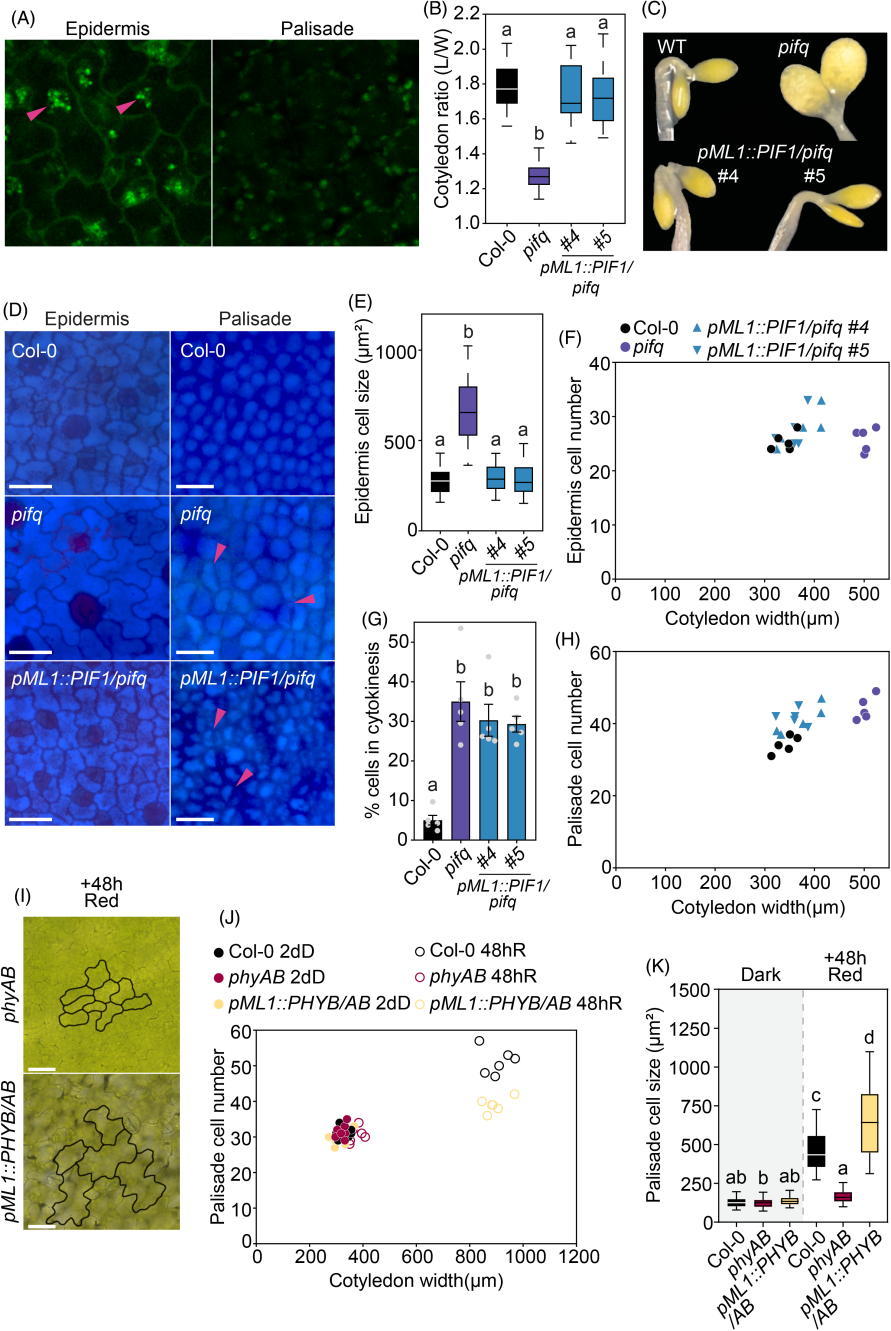
Epidermis‐specific PIF1 expression reverts the *pifq* photomorphogenic cotyledon phenotype in the dark but not the elevated cell division in the palisade. (A) *PIF1‐GFP* expression under the *pML1* promoter is detected by confocal microscopy in the nuclei of epidermis cells (magenta arrows) but not in the palisade. Seedlings were grown for 3 days in dark and incubated for 16 h in MG‐132 (50 μm). (B) Cotyledon ratio quantification of WT, *pifq*, and *pML1::PIF1‐GFP* lines (*#4* and #*5*) in 3‐day‐old dark‐grown seedlings (3dD). Data are means ± SEM of at least 25 seedlings. (C) Visual phenotypes of representative seedlings grown in (B). (D) Visual phenotypes of epidermis and palisade cells in WT, *pifq*, and *pML1::PIF1‐GFP #4/5* cotyledons grown in the dark for 2 days. (E) Epidermis cell size quantification in WT, *pifq*, and *pML1::PIF1‐GFP #4/5* seedlings grown in the dark for 2 days. Boxplots indicate the first quartile, median, and third quartile. Whiskers indicate 5–95 percentiles. (F) Representation of epidermis cell number relative to cotyledon width in WT, *pifq*, and *pML1::PIF1‐GFP #4/5* seedlings grown in the dark for 2 days. Each dot represents an individual cotyledon. (G) Percentage of palisade cells in visible division in WT, *pifq*, and *pML1::PIF1‐GFP #4/5* cotyledons grown in the dark for 2 days. Data are means ± SEM of at least six cotyledons. (B, E, G) Letters denote the statistically significant differences using one‐way anova followed by *post hoc* Tukey's test (*P* < 0.05). (H) Representation of palisade cell number relative to cotyledon width in WT, *pifq*, and *pML1::PIF1‐GFP #4/5* seedlings grown in the dark for 2 days. Each dot represents an individual cotyledon. (I) Visual phenotypes of epidermis cells in *phyAB* and *pML1::PHYB/AB* cotyledons grown in the dark for 2 days and transferred to red light for 48 h. (J) Representation of palisade cell number relative to width in WT, *phyAB* and *pML1::PHYB/AB* seedlings grown in the dark for 2 days (2dD) and transferred to red light (R) for 48 h. Each dot represents an individual cotyledon. (K) Palisade cell size quantification in WT, *phyAB* and *pML1::PHYB/AB* seedlings grown in the dark for 2 days (2dD) and transferred to red light (R) for 48 h. Data in boxplots indicate the first quartile, median, and third quartile of *n* ≥ 125 cells pooled from at least five individual cotyledons. Whiskers indicate 5–95 percentile. Letters denote statistically significant differences using two‐way anova followed by *post hoc* Tukey's test (*P* < 0.05).

To start to shed light on the molecular mechanisms underlying cotyledon cell expansion and division, we took into consideration a recent report showing that PIF4 activity in epidermal cells affected cotyledon separation during skotomorphogenesis but not hypocotyl elongation (Kim et al., 2020). These data prompted us to hypothesize that epidermal PIFs could trigger epidermis expansion in the dark that might be enough to drive palisade cell division and growth, and overall cotyledon development. To test this, we chose to focus on PIF1, as it has been described to be the major PIF contributor during skotomorphogenesis (Leivar et al., 2008; Pfeiffer et al., 2014). Transgenic plants expressing PIF1‐GFP under the control of the epidermis‐specific *MERISTEM LAYER1* (*ML1*) promoter were generated in the *pifq* mutant background, and PIF1‐GFP in these seedlings was detected in the epidermis pavement cells forming nuclear speckles, but not in the palisade (Figure 6A; Figure S3) confirming tissue‐specific accumulation. *PIF1* expression in the epidermis did not affect hypocotyl length and suppressed the cotyledon separation phenotype of *pifq* (Figure S4) in accordance with the reported PIF4 data (Kim et al., 2020). Additionally, PIF1 activity in the epidermal cells was sufficient to suppress the expanded cotyledon phenotype of *pifq* in the dark, both in terms of area (Figure 6B–E) and also cell morphology, which displayed the elongated epidermal cells characteristic of etiolated WT cotyledons (Figure 6D). Moreover, no difference in epidermis cell number was observed when comparing *pifq* and *pML1::PIF1‐GFP/pifq* (Figure 6F), in agreement with cotyledon expansion not requiring division in the epidermis. Strikingly, when examining the palisade, we observed that *pML1::PIF1‐GFP/pifq* maintained elevated division rates comparable to *pifq* despite the unexpanded cotyledons (Figure 6G). In fact, both lines (*pifq and pML1::PIF1‐GFP/pifq*) showed similar palisade cell number, causing *pML1::PIF1‐GFP/pifq* to lose linearity in the correlation between cotyledon width and palisade cell number (Figure 6H).

We also attempted to generate transgenic plants to express *PIF1* under a mesophyll‐specific promoter, an objective that recently proved to be challenging (Kim et al., 2020). We discarded the commonly used *CHLOROPHYLL A/B BINDING PROTEIN 3* (*CAB3*) or *IQ‐DOMAIN‐22* (*pIQD22*) promoters, as both have been recently shown to also drive epidermal activity in cotyledons, probably through leaky expression (Kim et al., 2020; Procko et al., 2022), and expressed *PIF1:mScarlet* under the control of the *SQUALENE MONOOXYGENASE 6* promoter (*pSQE6*), shown to drive expression in the palisade and the lowermost spongy layer in leaves (Procko et al., 2022). However, we were not able to detect *PIF1* expression in our transformed seedlings, probably due to very low expression levels in the cotyledon, as previously reported (Sun et al., 2016).

To further understand the epidermis/palisade interplay in the regulation of cotyledon expansion, we next examined available *pML1::PHYB‐GFP* expressing lines in the *phyAphyB* background (*pML1::PHYB‐GFP/AB*) (Kim et al., 2016). *phyB* expression in the epidermis was sufficient to drive cotyledon expansion in response to light (Figure 6I,J), showing the characteristic lobe morphology (Figure 6I). Strikingly, when examining the palisade, we observed that *pML1::PHYB‐GFP/AB* palisade cells were abnormally larger (Figure 6K), possibly due to the mechanical stress exerted by the growing epidermis and exhibited highly reduced cell division (Figure 6J).

Together, these results underscore the importance of the epidermis in cotyledon growth, suggesting that a phy/PIF cell‐autonomous activity in the epidermis can govern the extent of cotyledon expansion. Importantly, our results also highlight that the regulation of the palisade cell division does not need a light signal from the epidermis, although mechanical stress from an expanding epidermis can trigger some palisade cell division and both signals probably co‐exist. Our results strongly suggest that the light‐induced palisade division rate is cell‐layer autonomous and reveal a cell‐autonomous PIF activity in the palisade to repress cell division in the dark.

## DISCUSSION

**Figure 7:**
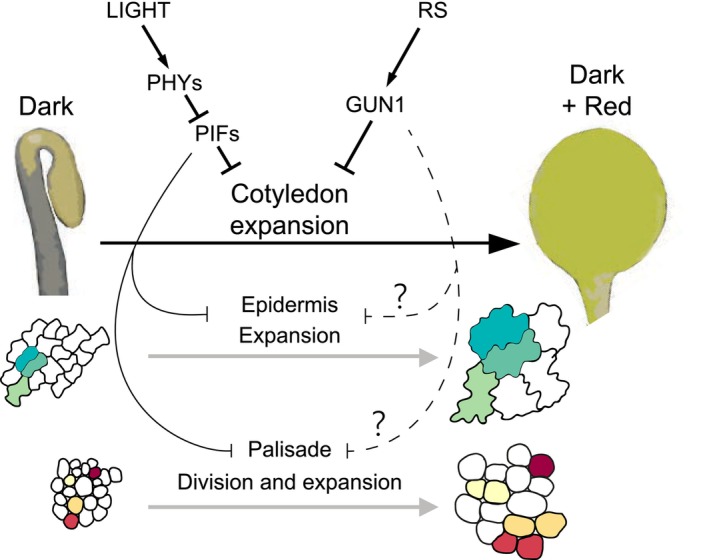
Model of PIF‐ and GUN1‐mediated regulation of cotyledon expansion during seedling deetiolation. Schematic model depicting PIF‐mediated repression of cotyledon expansion in the dark. Upon exposure to light, active phytochromes repress PIF activity and trigger PIF degradation, and cotyledons start to rapidly expand. Cotyledon growth involves epidermal cell expansion and palisade cell division and expansion. Cotyledon growth takes place coinciding with etioplast‐to‐chloroplast development. If chloroplast biogenesis is disrupted, GUN1‐mediated retrograde signaling inhibits cotyledon expansion. Dashed lines and question marks indicate currently unknown connections.

We have shown here that light‐induced cotyledon expansion involves a switch in the growth direction that is antagonistically regulated by light through the PIF/phy system and by GUN1‐mediated retrograde signaling. Our work examines the contribution of cell expansion and division in the epidermis and the mesophyll, and shows for the first time that cotyledon palisade expansion involves cell division. This aligns with the enrichment of cell division‐related genes antagonistically regulated by the PIF/phy system and RS specifically in the cotyledon. Furthermore, using mutant lines specifically expressing PIF1 and phyB in the epidermis, our results show that epidermis expansion can drive cell growth in the palisade, whereas mesophyll cell division is mostly regulated by light at the tissue‐specific level. These results, together with previous data, support a model whereby cotyledon expansion during deetiolation relies on a PIF/phy‐regulated switch in growth direction from longitudinal in the dark to transversal in the light, and involves distinct growth strategies for the epidermis (based on cell expansion) and mesophyll layers (based on cell expansion and division) (Figure 7). Light‐induced cotyledon growth requires and coincides temporarily with etioplast‐to‐chloroplast development, which ensures the availability of the maximum light‐capturing surface for photosynthesis when the light intensity is adequate. The system is exquisitely sensitive to the prevailing light environment, and potentially damaging high light conditions activate GUN1‐mediated RS to inhibit cotyledon expansion and reduce the exposed area susceptible to damage (Figure 7). Hence, RS acts as a developmental brake that could be critical under conditions such as fluctuating light intensity under a canopy with locally high irradiance sunflecks (Durand et al., 2021), or as a function of cloud coverage (Schneider et al., 2019).

We have established a novel framework to study how cotyledon expansion is regulated during seedling deetiolation. Cotyledon expansion has been traditionally overlooked in light signaling and seedling de‐etiolation research, where hypocotyl elongation and cotyledon separation to a lesser extent have been extensively and preferentially used as phenotypic readouts. The advantages of hypocotyl as a simple model, thanks to its unidirectional growth based on cell expansion (Gendreau et al., 1997) and the similar morphology of its cell types, have contributed greatly to the advancement of our knowledge in light signaling (Zhao et al., 2020), de‐etiolation (Jedynak et al., 2021), light‐hormonal interplay (Zhang et al., 2015), circadian clock (Martín et al., 2020), and shade avoidance (Sharma et al., 2023) among others. However, the complexity of seedling development has long been evident in the opposite effect of light on hypocotyl and cotyledon development, where light inhibits hypocotyl elongation while promoting the expansion of cotyledons (Bou‐Torrent et al., 2008). This opposite regulation is crucial for seedling survival, and a detailed understanding of cotyledon development can provide insights into the underlying mechanisms.

We have shown that light‐induced cotyledon expansion relies on a switch of organ growth direction, from longitudinal to transversal (Figure 1). Because plant cells are enclosed by a rigid cell wall and cannot migrate, the control of directional cell growth determines morphogenesis and organ geometry. This establishment and coordination of plant growth and development relies on both mechanical as well as biochemical signals governing cell growth (Jonsson et al., 2022). The regulation of growth direction remains relatively unclear, but it is in part controlled by the orientation of the cortical microtubules (Kirchhelle et al., 2019). Interestingly, some of the enriched gene‐ontology terms found in our transcriptomic analysis of the light/PIF‐induced and RS‐regulated genes in the cotyledon (Figure 3) are related to microtubule movement and organization. A recent report has shown that mutants in the OVATE FAMILY PROTEIN (OFP) of transcriptional repressors exhibit long cotyledons when grown in the light due to enhanced cell elongation (Zhang, Wu, et al., 2020). OFP proteins regulate cell growth direction in cotyledons by interacting with TONNEAU2 (TON2) to modulate microtubule orientation. TON2 is able to interact with LONGIFOLIA (LNG) proteins (Drevensek et al., 2012), which positively regulate polar cell elongation in the leaf length axis to determine morphology, and have been shown to be directly regulated by PIFs (Hwang et al., 2017). Interestingly, the OFP‐TON2 interaction is mediated by brassinosteroid levels (Zhang, Wu, et al., 2020), and LNGs promote growth by activating the auxin pathway (Hwang et al., 2017). Growth directionality in several organs is determined by hormone gradients. In apical hooks, the PIN3 and PIN4 auxin carriers drive auxins toward the outermost convex side of the hook, draining auxin concentration in the inner concave side and promoting hook opening (Abbas et al., 2013; Žádníkova et al., 2010). Hook opening is also controlled by PIF‐regulated cytokinesis and microtubule orientation, although it is still unclear how this may relate to auxin distribution (Zhang et al., 2021). In roots, auxin gradients control root growth, cytokinin acts antagonistically to regulate cell division, and gibberellins delimit the front between cell expansion and cell division (Achard et al., 2009; Beemster & Baskin, 2000; Grieneisen et al., 2007). Additionally, cytokinin gradients generate asymmetric cell division to generate root bending in hydrotropic responses (Chang et al., 2019). In leaves, WOX proteins (WUSCHEL‐RELATED HOMEOBOX) regulate auxin biosynthesis in leaf margins, creating an auxin gradient that promotes leaf width‐specific growth (Zhang, Runions, et al., 2020). In all, the connection between organ growth directionality, cell elongation, microtubule orientation, and hormone pathways and/or gradients has been extensively studied in the regulation of leaf shape, hypocotyl elongation, apical hook development or root growth (Jonsson et al., 2022). However, whether cotyledon expansion relies on a similar regulatory coordination of mechanical and hormone signals and gradients is unknown. Our finding that hormone categories (such as brassinosteroid, ABA and ethylene) are enriched in our transcriptomic analysis of the light/PIF‐ and RS‐regulated genes during cotyledon expansion together with microtubule terms (Figure 3C) suggests a similar general regulatory framework. Interestingly, all three hormones have been previously shown to affect microtubule orientation (Hamant & Traas, 2010). In addition, organ‐specific transcriptomic analysis identified the auxin‐regulated *Small Auxin Up RNA* (*SAUR*) genes whose transcripts are light‐induced in cotyledons and/or repressed in hypocotyls (Sun et al., 2016). Importantly, PIFs were shown to directly bind these *SAURs* and differentially regulate their expression in cotyledons and hypocotyls possibly through interaction with organ‐specific cofactors (Sun et al., 2016), suggesting overall that auxin might play a role in the regulation of cotyledon expansion. Whether auxin and/or SAURS might affect microtubule orientation is currently unknown. A more recent organ‐specific transcriptomic analysis (Zhang et al., 2021) identified the PIF direct target BUB3.1, important in the control of hook opening through cytokinesis regulation. Future studies to explore the mechanical and biochemical signaling interplay in cotyledons will be of great interest, as well as to determine whether there is a tissue‐level control differentiating cell growth direction of epidermis and palisade cells, for which the cotyledon provides an excellent study system.

The effect of light during seedling deetiolation on the shape of epidermal and palisade cells is clearly distinct. Epidermal cells acquire lobed puzzle‐shaped forms as they expand (Figure 4). This complex shape has been proposed to reduce mechanical stress in the cell wall during isotropic growth and prevent bulging due to turgor pressure (Sapala et al., 2018). In contrast, cells in the palisade layer expand, keeping the original rectangular shape (Figure 5). Interestingly, our finding that palisade cells undergo cell division during cotyledon expansion might also represent a strategy to minimize and cope with mechanical stress. Indeed, the orientation of cell division has been proposed to align with the direction of maximal mechanical stress to produce smaller cells that will have a reinforced cell wall in the orientation of maximum tension (Robinson, 2021). In addition, although the regulation of chloroplast division and the integration with cell division and expansion is still poorly understood (Jarvis & López‐Juez, 2013), we propose that cell division in the palisade may indirectly allow an increase in the number of chloroplasts in the mesophyll. This would contribute to increase the photosynthetic capacity of the expanding cotyledon during seedling deetiolation, although to the best of our knowledge, no studies have addressed this directly.

Our results show that the light regulation of palisade cell division seems to be largely independent of the epidermis, opening the question of how these two layers coordinate growth during cotyledon expansion. In our experiments expressing PIF1 and phyB under the control of the epidermis‐specific promoter pML1, epidermis and palisade extension are coordinated (Figure 6). It seems, therefore, that the mechanical force exerted by the epidermis works as a coordinating signal for the palisade, as suggested in previous work (Verger et al., 2018). However, our finding that cell division in the palisade largely escapes this coordination suggests that the regulation of cell expansion and division is likely uncoupled, with cell division being more strictly regulated by light at a cell‐type specific level.

Finally, our work highlights the use of cotyledon development in response to light as a new model system to study organ shape regulation, a fundamental challenge in biology. Cotyledons provide a powerful model to address unanswered questions for which other organs like hypocotyls are not suitable, such as the coordination of different cell layers during growth, light‐induced multidimensional expansion, or the coordination of the individual axes in growth. Moreover, although cotyledon expansion and leaf growth present considerable differences (for example the presence of a leaf primordia that initially grow by cell division and is progressively substituted by cell expansion), leaf and cotyledon cell layer structures are equivalent, and cotyledons can help address open questions in leaf development such as the interconnection between division and expansion or the interplay between the different cell layers (Gonzalez et al., 2012), using the tools and approaches described here.

## EXPERIMENTAL PROCEDURES

### Plant material

*Arabidopsis thaliana* seeds used in this manuscript include the previously described Col‐0, *phyA‐211* (Reed et al., 1994), *phyB‐9* (Reed et al., 1993), *phyA‐211phyB‐9* (Reed et al., 1994), *pifq* (Leivar et al., 2008), *cop1‐6* (McNellis et al., 1994), and *pML1::phyB‐GFP/phyAphyB* (*pML1::PHYB‐GFP/AB*; Kim et al., 2016). To generate the *pML1::PIF1‐GFP* transgenic lines, the *MERISTEM LAYER‐1* promoter described in van Es et al. (2018) was amplified using primers indicated in Table S2 (EMP1730 + EMP1731) and cloned into pDONR‐P4‐P1R using the BP recombination reaction (Gateway; Invitrogen). Similarly, the *PIF1* coding sequence was amplified (using primers EMP1732 + EMP1732, see Table S2) and cloned into pDONR221 and the GFP coding sequence into pDONR‐P2R‐P3. LR recombination reaction (Gateway®; Invitrogen, Waltham, MA, USA) was performed to generate in‐frame *pML1::PIF1‐GFP* constructs into the pH7m34GW expression vector that was transformed into the *pifq* background.

### Growth conditions

Seeds were surface sterilized with 20% bleach and 0.5% sodium dodecyl sulfate (SDS) for 10 min and rinsed five times with distilled sterile water. Seeds were plated on half‐strength Murashige and Skoog (0.5 × MS) medium without sucrose, stratified at 4°C in the dark for 4 days, and exposed to white light (100 μE) at 21°C for 3 h to induce germination. Seedlings were then kept in the dark at 21°C for 2 days and transferred to continuous red light (20 μE) at 21°C for the specified hours in each experiment. For dark‐extended experiments, seedlings were kept in the dark at 21°C for 2, 3, 4, or 5 days. For lincomycin treatments, the medium was supplemented with 0.5 mm lincomycin (Sigma, St.Louis, MO, USA; L6004) (Sullivan & Gray, 1999). Red light was provided by (PHILIPS, Amsterdam, Netherlands) GreenPower research module in deep red (maximum at 660 nm). Light intensity was measured with a built‐in LI‐COR (Lincoln, NE, USA) LI‐190R Quantum Sensor in the red growth chambers (Aralab, Rio de Mouro, Portugal) and with a hand‐held SpectraPen mini (Photon Systems Instruments, Drásov, Czech Republic) for the white light growth chamber (Aralab, Rio de Mouro, Portugal).

### Cotyledon imaging measurements

To quantify cotyledon expansion, seedlings were gently pushed against the media, and cotyledons were gently separated using tweezers and photographed using a digital camera (Nikon; Tokyo, Japan, D7000). Measures were performed using NIH image software (Image J, National Institutes of Health). The length (from the cotyledon base at the petiole attachment point to the cotyledon tip in a straight line) and the width (from one lateral border to the other in a straight line in the wider point of the cotyledon) were measured. Only one cotyledon per seedling was measured. The cotyledon ratio was defined by dividing length by width.

### Cell imaging and measurements

Cell imaging was performed using the DM6 epifluorescence microscope (Leica, Hesse, Germany). Cotyledons were cut and mounted in water. Multiple images from each cotyledon were obtained using a 63× lens and later merged to generate composite images of the whole cotyledon using the LASX software (Leica, Hesse, Germany). For dark, 6, and 12 h of red light exposure, cotyledon images were captured using the UV excitation range A‐filter cube (340–380 nm) and a suppression filter LP425, providing high contrast to the cell walls without the need for chemical staining. For 6 and 12 h red light images, the “red channel” from the images was deactivated to eliminate the chlorophyll signal. In 24 and 48‐h red light samples, images were captured using bright field imaging, as high chlorophyll content interferes with the signal obtained with the A‐filter cube.

For cell size (epidermis and palisade) measurements, at least 25 cells per cotyledon in at least five cotyledons (*n* ≥ 125) were measured from the central part of the cotyledon. In the palisade, only non‐dividing cells were measured. Measures were performed using NIH image software (Image J, National Institutes of Health). For cell counting, a straight line following the width axis in the middle of the cotyledon was drawn, and all cells crossed by the line were counted. For the quantification of the percentage of palisade cells in cytokinesis, the total number of cells and those in visible cytokinesis were counted in an area of 50 × 10^3^ μm^2^ in the center of the cotyledon.

### Fluorescence microscopy

For PIF1‐GFP epidermal detection, *pML1::PIF1‐GFP* seedlings were grown for 3 days in the dark. Then, seedlings were incubated in the dark for 16 h in liquid MS media with MG‐132 (50 μm) (Calbiochem, San Diego, CA, USA) protease inhibitor. Lines were visualized using the SP5 confocal microscope (Leica, Hesse, Germany) with an excitation argon laser (488 nm) and emission range (500–600 nm).

### Statistical analysis

Differences in cotyledon ratio, percentage of cells, and cell size between two genotypes were analyzed by Student's *t*‐test, and significantly different pairs (*P* < 0.05) were represented by asterisks. To identify differences in cotyledon ratio, cell size, percentage of cells, or relative gene expression between multiple genotypes in one light condition, data were analyzed using one‐way anova followed by *post hoc* Tukey's test (*P* < 0.05), and results were represented by letters. To identify differences in cotyledon ratio or cell size between multiple genotypes and light conditions, data were analyzed using two‐way anova followed by *post hoc* Tukey's test (*P* < 0.05), and results were represented by letters.

### Gene expression analyses

To detect *PIF1* levels in the generated *pML1::PIF1‐GFP* lines, qRT‐PCR was performed as described previously (Veciana et al., 2022). Briefly, seedlings were grown in the dark, and total RNA was extracted using Mawxell RSC plant RNA Kit (Promega, Madison, WI, USA). One microgram of total RNA was treated with DNase I (Promega) according to the manufacturer's instructions. First‐strand cDNA synthesis was performed using the NZYtech First‐strand cDNA Synthesis Kit (NZYtech, Lisboa, Portugal), and 2 μl of 1:25 diluted cDNA with water was used for real‐time PCR (LightCycler 480; Roche, Basel, Switzerland) using SYBR Premix Ex Taq (Takara, Kusatsu, Shiga Prefecture, Japan) and primers at 300 nm concentration. Gene expression was measured in three independent biological replicates, and at least two technical replicates were done for each of the biological replicates. *PP2A* (AT1G13320) was used for normalization (Shin et al., 2007). Primer sequences are described in Table S2.

### Definition of “Light UP/DOWN Cot” and “Light UP/DOWN Cot + Hyp” gene sets

To establish genes regulated by light in cotyledons but unaffected by light in hypocotyls, or those that exhibit opposite expression patterns in these two organs, we used the gene classification established by Sun et al. (2016). Supplementary dataset 1 from Sun et al. (2016; “Light responsive genes at 1 and 6 h in cotyledons and hypocotyls”) includes genes that are light‐regulated in cotyledons and/or hypocotyls, with a log_2_(FC) > |1| and with a p‐value < 0.05. From the 8889 genes identified as light regulated at any time point (1 or 6 h) or organ, we discarded 130 genes that displayed an inconsistent direction of light regulation when comparing the two times points. We then focused on the 5760 genes regulated by light in the cotyledons, while the remaining 2999 were regulated by light only in hypocotyls. We further classified the 5760 genes as “Light UP Cot” (2122 genes, upregulated by light in cotyledons, 1810 cotyledon‐specific and 312 also downregulated in hypocotyls), “Light UP Cot + Hyp” (1178 genes, upregulated by light in both cotyledons and in the hypocotyl), “Light DOWN Cot” (1781 genes, downregulated by light in cotyledons, 1781 1640 cotyledon specific and 141 also upregulated in hypocotyls), and “Light DOWN Cot + Hyp” (679 genes, downregulated by light in both cotyledons and in the hypocotyl).

### RNAseq quantification of gene expression levels, heatmap, and gene ontology analyses

Total mRNA levels for the “Light UP/DOWN Cot” and “Light UP/DOWN Cot + Hyp” genes in WT and *pifq*, as well as for the NF‐treated WT and *gun1*, were obtained by re‐quantification of public sequencing data (GSE79576; Sun et al., 2016 and GSE110125; Zhao et al., 2019, respectively) with *vast‐tools* v2.5.1 (Martín et al., 2021). This tool provides the corrected‐for‐mappability RPKMs (cRPKMs) (number of mapped reads per million mapped reads, divided by the number of uniquely mappable positions of the transcript) (Labbé et al., 2012). Re‐quantification ensured homogeneity of both experiments (Sun et al., 2016; Zhao et al., 2019) for comparison, and re‐quantified total mRNA levels were used in Figure 3(A,B). Heatmaps in Figure 3(B) depict *Z* score of fold‐change expression and were generated using the heatmap.2 function from the gplots package in R (https://search.r‐project.org/CRAN/refmans/gplots/html/heatmap.2.html).

For the Gene Ontology analysis in Figure 3(C) and Figure S1, genes in the “Light Cot” and “Light Cot + Hyp” subsets were filtered for previously defined PIFq‐regulated in the dark (Sun et al., 2016) and RS‐regulated in the light (Zhao et al., 2019). About 10% of the “Light UP Cot” gene set (219 of 2122 genes) and 6.5% of the “Light DOWN Cot” (117 of 1781 genes) met both criteria (Table S1). Significantly enriched biological processes and molecular functions among the different sets of genes were defined using the functional annotation classification system DAVID (Huang et al., 2007).

## ACCESSION NUMBERS

Raw sequencing data used in this study were obtained from the Sequence Read Archive (accession numbers GSE79576 and GSE110125).

## CONFLICT OF INTEREST

The authors declare no conflicts of interest.

## Supporting information


**Figure S1.** Gene Ontology (GO) enrichment of the “Light UP Cot + Hyp” and “Light DOWN Cot + Hyp” subsets regarding biological process and molecular function categories.

**Figure S2.** Epidermis cell size and palisade cell division phenotype of *cop1*. (a) Quantification of epidermis cell size in WT and *cop1‐6* in 2‐day dark‐grown seedlings (2dD). Data in boxplots indicate the first quartile, median, and third quartile of *n* ≥ 125 cells pooled from at least five individual cotyledons. Whiskers indicate 5–95 percentiles. Statistical differences relative to WT in each timepoint are indicated by an asterisk (Student's *t*‐test. *P* < 0.05). (b) Visual phenotypes of Col‐0 and *cop1‐6* epidermis cells. (c) Percentage of palisade cells with a visible division plane in WT and *cop1‐6* in 2‐day dark‐grown seedlings (2dD). Data indicate SEM of five cotyledons. Asterisks indicate statistically significant differences (Student's *t*‐test. *P* < 0.05). (d) Visual phenotypes of WT and *cop1‐6* palisade cells.

**Figure S3.** Epidermis‐specific *PIF1‐GFP* characterization of independent *pML1::PIF1‐GFP/pifq* lines. (a) Quantification of cotyledon ratio (length/width) in WT, *pifq*, and independent *pML1::PIF1‐GFP/pifq* transgenic lines. Data in boxplots indicate the first quartile, median, and third quartile of *n* ≥ 20 seedlings. Whiskers indicate 5–95 percentile. Letters denote the statistically significant differences using one‐way ANOVA followed by *post hoc* Tukey's test (*P* < 0.05). (b) *PIF1* expression relative to Col‐0 set at 1 in 2dD Col‐0, *pifq*, and independent *pML1::PIF1‐GFP/pifq* transgenic lines. Data are the means ± SE of biological triplicates (*n* = 3). (c) Detection of *PIF1‐GFP* accumulation by confocal microscopy in the nuclei of epidermis cells (magenta arrows). Seedlings were grown for 3 days in the dark and incubated for 16 h in MG‐132 (50 μm).

**Figure S4.** Epidermis‐specific *PIF1‐GFP* expression complements the constitutively photomorphogenic cotyledon phenotype of *pifq* in *pML1::PIF1‐GFP/pifq* lines but not the short hypocotyl. (a) Expression of epidermis‐specific *PIF1‐GFP* does not rescue the short hypocotyl phenotype of *pifq*. Hypocotyl length of 3 dD WT, *pifq*, and two independent *pML1::PIF1‐GFP/pifq* lines. Data in boxplots indicate the first quartile, median, and third quartile of *n* ≥ 20 seedlings. Whiskers indicate 5–95 percentile. Letters denote the statistically significant differences using one‐way anova followed by *post hoc* Tukey's test (*P* < 0.05). (b) Visual phenotypes of representative seedlings grown as in (a), showing complementation of the cotyledon expansion and separation phenotype.

**Table S1.** Gene lists corresponding to the light‐regulated gene sets defined in Figure 3. These genes were identified based on their light‐responsiveness in cotyledon and hypocotyl organs and their regulation by PIFs (Sun et al., 2016) and retrograde signaling (RS; Zhao et al., 2019).

**Table S2.** Primer sequences used for cloning and gene expression analysis.

## Data Availability

All relevant data can be found within the published article and its supporting material.
