# Supplementary figures and images for: A PIF‐regulated switch in cell axis growth drives cotyledon expansion through tissue‐specific cell expansion and division
Source: Plant J. 2025 May 16;122(4):e70196. doi: 10.1111/tpj.70196 (PMC12083523; doi:10.1111/tpj.70196)

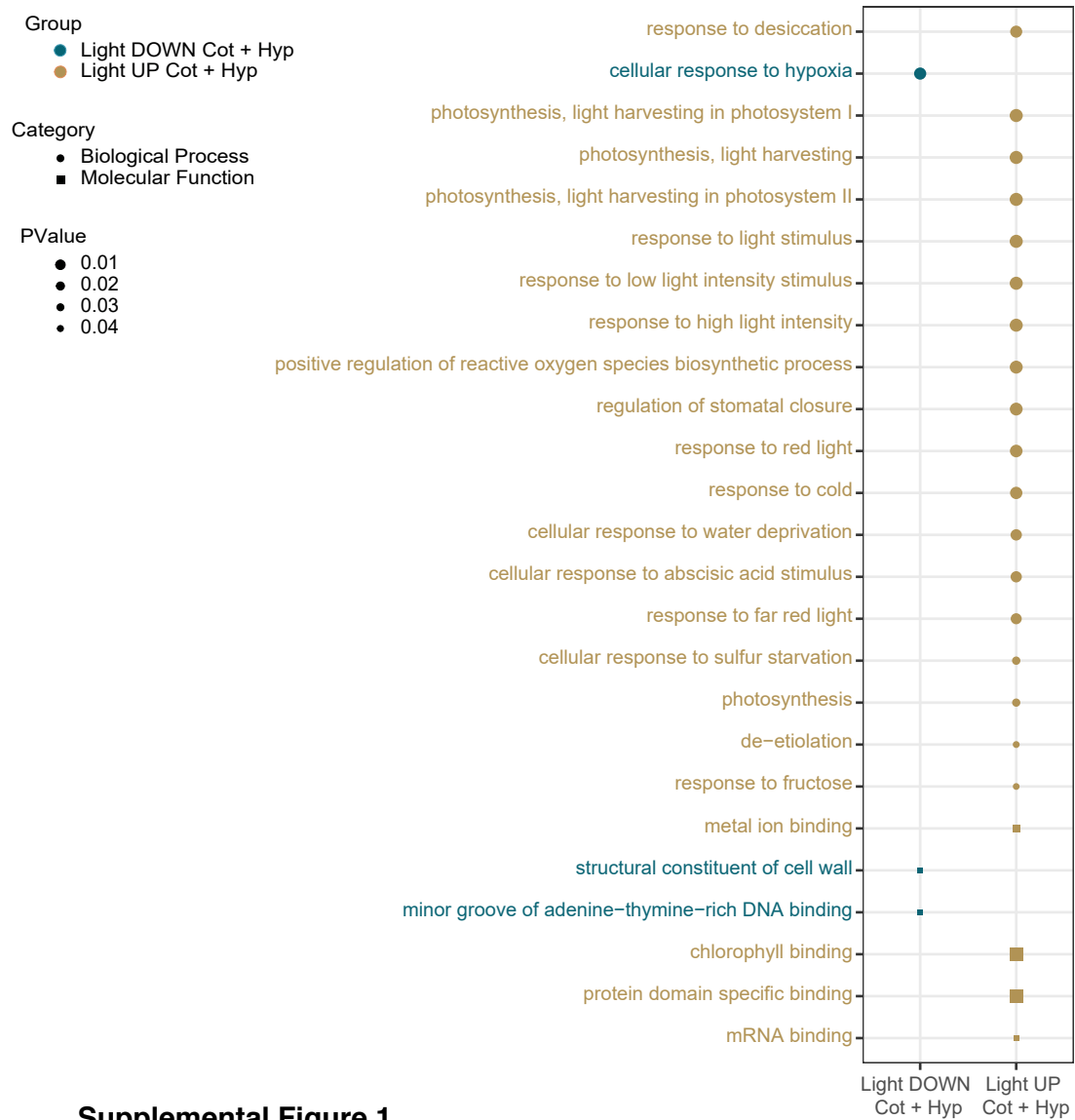

**Supplemental Figure 1**

Supplement: Supplementary file 1 — Figure S1. Gene Ontology (GO) enrichment of the “Light UP Cot + Hyp” and “Light DOWN Cot + Hyp” subsets regarding biological process and molecular function categories. [file TPJ-122-0-s004.pdf]

## Supplemental Figure 2

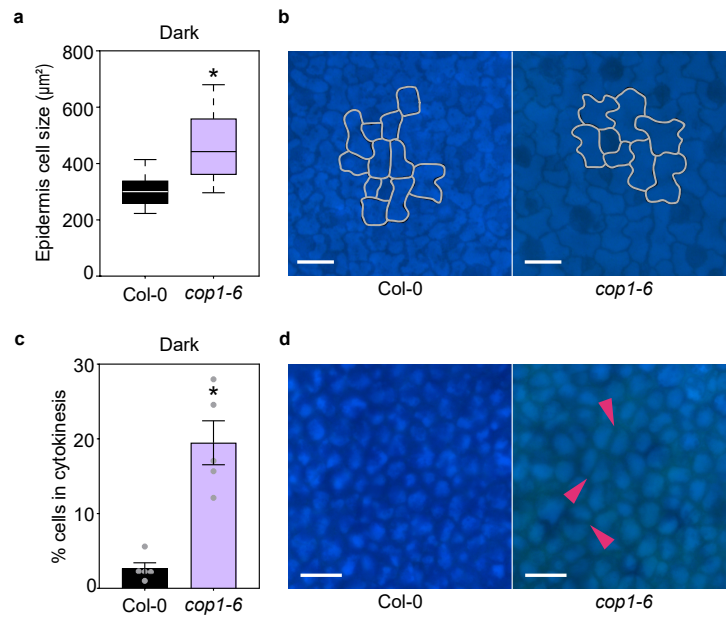

Supplement: Supplementary file 2 — Figure S2. Epidermis cell size and palisade cell division phenotype of cop1. (a) Quantification of epidermis cell size in WT and cop1‐6 in 2‐day dark‐grown seedlings (2dD). Data in boxplots indicate the first quartile, median, and third quartile of n ≥ 125 cells pooled from at least five individual cotyledons. Whiskers indicate 5–95 percentiles. Statistical differences relative to WT in each timepoint are indicated by an asterisk (Student's t‐test. P < 0.05). (b) Visual phenotypes of Col‐0 and cop1‐6 epidermis cells. (c) Percentage of palisade cells with a visible division plane in WT and cop1‐6 in 2‐day dark‐grown seedlings (2dD). Data indicate SEM of five cotyledons. Asterisks indicate statistically significant differences (Student's t‐test. P < 0.05). (d) Visual phenotypes of WT and cop1‐6 palisade cells. [file TPJ-122-0-s003.pdf]

# Supplemental Figure 3

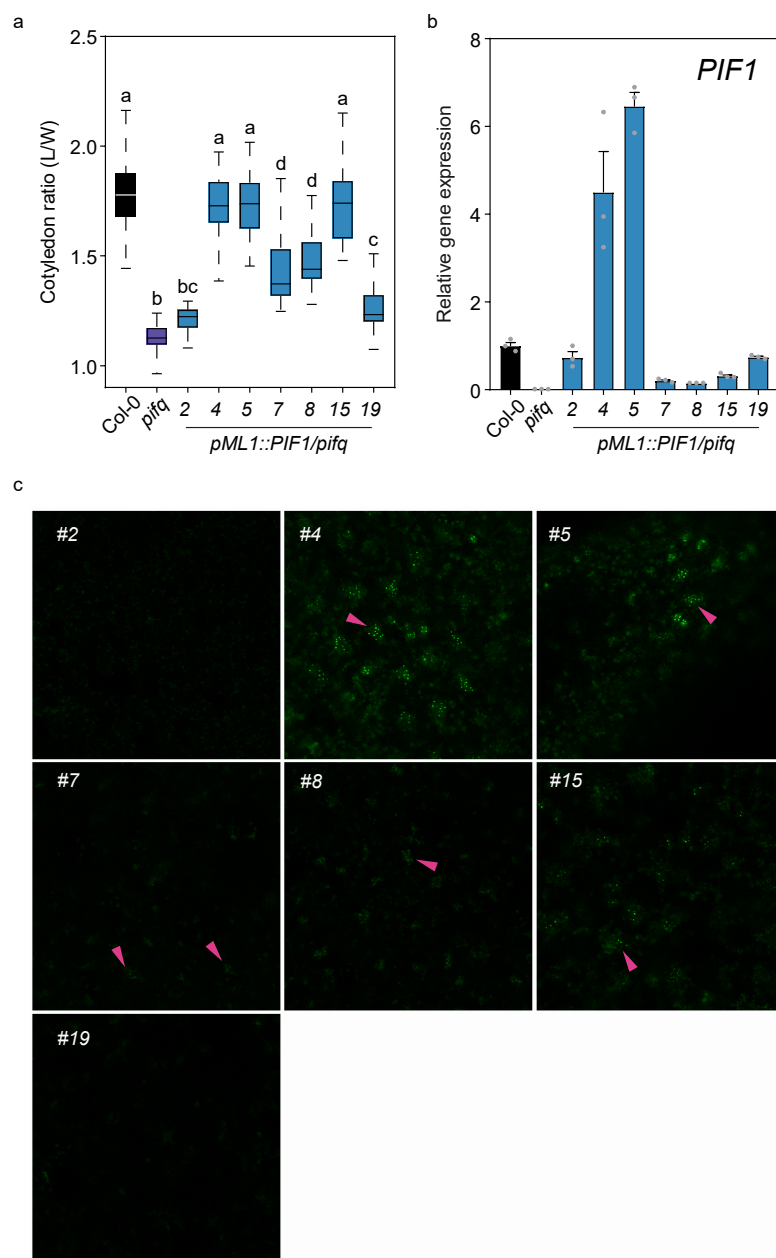

Supplement: Supplementary file 3 — Figure S3. Epidermis‐specific PIF1‐GFP characterization of independent pML1::PIF1‐GFP/pifq lines. (a) Quantification of cotyledon ratio (length/width) in WT, pifq, and independent pML1::PIF1‐GFP/pifq transgenic lines. Data in boxplots indicate the first quartile, median, and third quartile of n ≥ 20 seedlings. Whiskers indicate 5–95 percentile. Letters denote the statistically significant differences using one‐way ANOVA followed by post hoc Tukey's test (P < 0.05). (b) PIF1 expression relative to Col‐0 set at 1 in 2dD Col‐0, pifq, and independent pML1::PIF1‐GFP/pifq transgenic lines. Data are the means ± SE of biological triplicates (n = 3). (c) Detection of PIF1‐GFP accumulation by confocal microscopy in the nuclei of epidermis cells (magenta arrows). Seedlings were grown for 3 days in the dark and incubated for 16 h in MG‐132 (50 μm). [file TPJ-122-0-s002.pdf]

## Supplemental Figure 4

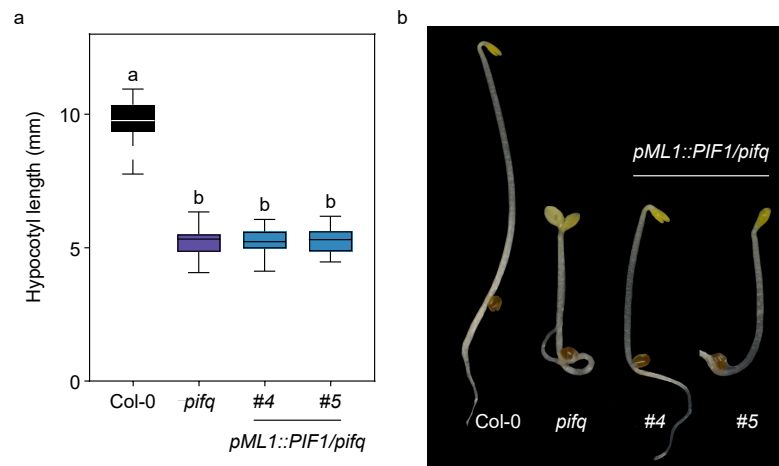

Supplement: Supplementary file 4 — Figure S4. Epidermis‐specific PIF1‐GFP expression complements the constitutively photomorphogenic cotyledon phenotype of pifq in pML1::PIF1‐GFP/pifq lines but not the short hypocotyl. (a) Expression of epidermis‐specific PIF1‐GFP does not rescue the short hypocotyl phenotype of pifq. Hypocotyl length of 3 dD WT, pifq, and two independent pML1::PIF1‐GFP/pifq lines. Data in boxplots indicate the first quartile, median, and third quartile of n ≥ 20 seedlings. Whiskers indicate 5–95 percentile. Letters denote the statistically significant differences using one‐way anova followed by post hoc Tukey's test (P < 0.05). (b) Visual phenotypes of representative seedlings grown as in (a), showing complementation of the cotyledon expansion and separation phenotype. [file TPJ-122-0-s006.pdf]
