# Supplementary material for: A PIF‐regulated switch in cell axis growth drives cotyledon expansion through tissue‐specific cell expansion and division
Source: Plant J. 2025 May 16;122(4):e70196. doi: 10.1111/tpj.70196 (PMC12083523; doi:10.1111/tpj.70196)
Supplement: Supplementary file 6 — Table S2. Primer sequences used for cloning and gene expression analysis. [file TPJ-122-0-s005.docx]

**Supplemental Table 2**

Primer sequences used for cloning and gene expression analysis.

| **Gene** | **AGI** | **Primer** | **Sequence 5’ to 3’** | **Description** |
| --- | --- | --- | --- | --- |
| *pML1* | *AT4G21750* | EMP1730 | GGGGACAACTTTGTATAGAAAAGTTGCAGCTTATCAAAGAAAAAACAAGAAC | Gateway cloning B4 |
| *pML1* | *AT4G21750* | EMP1731 | GGGGACTGCTTTTTTGTACAAACTTGCTAACCGGTGGATTCAGGGAGTTTCTTTA | Gateway cloning B1R |
| *PIF1* | *AT2G20180* | EMP1732 | GGGGACAAGTTTGTACAAAAAAGCAGGCTATGCATCATTTTGTCCCTGACTTCG | Gateway cloning B1 |
| *PIF1* | *AT2G20180* | EMP1733 | GGGGACCACTTTGTACAAGAAAGCTGGGTGACCTGTTGTGTGGTTTCCG | Gateway cloning B2 |
| *PIF1* | *AT2G20180* | EMP540 | ATCCAACCTCGGGCCAGCCT | qPCR |
| *PIF1* | *AT2G20180* | EMP541 | TTGGGTCGGGTGGAGACCGC | qPCR |
| *PP2A* | *AT1G13320* | EMP338 | TATCGGATGACGATTCTTCGT | qPCR |
| *PP2A* | *AT1G13320* | EMP339 | GCTTGGTCGACTATCGGAATG | qPCR |
